# Gut-derived metabolites as treatment targets in chronic kidney disease—an avenue toward personalized medicine

**DOI:** 10.1007/s00467-024-06609-w

**Published:** 2025-01-16

**Authors:** Johannes Holle, Hendrik Bartolomaeus

**Affiliations:** 1 Department of Pediatric Gastroenterology, Nephrology and Metabolic Diseases, Charité – Universitätsmedizin Berlin, Berlin, Germany; 2 Experimental and Clinical Research Center, a Cooperation of Charité – Universitätsmedizin Berlin and Max Delbrück Center for Molecular Medicine, Berlin, Germany; 3 German Centre for Cardiovascular Research (DZHK), Partner Site Berlin, Berlin, Germany; 4 Max Delbrück Center for Molecular Medicine in the Helmholtz Association, Berlin, Germany; 5 Institute of Experimental Biomedicine, University Hospital Würzburg, Würzburg, Germany

## Uremic toxins and the gut microbiome in chronic kidney disease

Chronic kidney disease (CKD) represents a hidden epidemic that is projected to become the fifth highest cause of years of life lost by 2040 [1]. The progressive loss of kidney function results in the retention of various uremic toxins in the systemic circulation. These toxins gain increasing importance as key mediators not only of the progression of CKD but also of its systemic complications, such as cardiovascular and mineral bone disease. Recent analyses of circulating metabolite profiles in two of the largest CKD cohorts—the Chronic Kidney Disease in Children (CKiD) cohort [2] and the German CKD cohort (GCKD) [3]—have advanced our understanding of the condition. Using standardized commercially available high-throughput mass spectrometry-based methods for metabolite detection and annotation (Metabolon Inc., Durham, USA), 1416 plasma metabolites have been measured in adult CKD patients [3]. There is, however, a pressing need for more information on the potentially pathogenic interplay of these metabolites with various organ systems in the context of health to CKD to frailty transition.

In this issue of *Pediatric Nephrology*, Ebrahimi and colleagues further analyze the CKiD metabolome data in a subgroup of 150 patients, focusing on five gut-derived metabolites to establish associations between metabolite accumulation and kidney function, disease etiology, and clinical outcomes [4]. The authors use targeted absolute quantification to pave the road toward clinical application.

There is flourishing evidence about the pivotal role of the gut microbiome in regulating various facets of health and disease [5]. This ecosystem harbors up to 150 times more genes than the human genome [6], many of which are crucial for the bacterial breakdown of mammalian diets. CKD leads to an accumulation of circulating microbiome-derived metabolites in both animal models [7] of CKD and patients with CKD [8]. In CKD, alterations in the composition and function of the gut microbiome have been described in adults [9] and children [10]. Gut microbiome alterations are due to multiple factors like nutrition, the overgrowth of urease-producing bacteria due to high urea levels, or drug intake. These alterations contribute not only to the increased production of uremic toxins but also disrupt intestinal wall integrity, facilitating the entry of these solutes into the systemic circulation [11]. The pivotal contribution of the gut microbiome in the accumulation of uremic toxins, in addition to reduced renal clearance, has been highlighted in recent animal experiments and translational studies. The transfer of the gut microbiome from CKD patients into germ-free mice has direct implications on metabolite accumulation and clinical endpoints compared to the transfer of the gut microbiome from healthy donors [9]. Moreover, key enzymes for microbial generation of prototypic uremic toxins have been shown to be highly abundant in fecal samples from CKD donors when compared to healthy donors using qPCR-based methods [10, 12].

Based on the knowledge about the microbial origin of these metabolites, the gut microbiome becomes very attractive as a target to treat by dietary interventions like a high-fiber diet and different biotics designed to have beneficial effects on the host, including prebiotics (nonviable substances modulating the microbiome), probiotics (live microorganisms), postbiotics (bacterial metabolic products), and synbiotics (combination of pre- and probiotics) [13].

## The need for more pediatric research in the field of microbiome-host interaction

It is worth mentioning that clinical and molecular phenotypes in adult patients with CKD are highly influenced by confounding comorbidities, cumulating in multimorbidity and frailty, especially in the elderly population with CKD [1]. Many of these CKD-related factors have been shown to have an important impact not only on the composition and the metabolic function of the gut microbiome [14], but also on disease outcomes. Therefore, analyzing microbiome-host interactions in pediatric populations, allows more disease-specific insights into CKD pathophysiology, as confounding comorbidities are less frequent compared to adult populations.

However, children also exhibit unique properties not only in terms of microbiome maturation and development [15] but also in terms of metabolite generation and handling as a consequence of pubertal maturation, a larger body water volume in young children, lower circulating protein abundances, a different pattern of underlying kidney disease, and subsequently altered solute handling at the proximal tubule [16]. Moreover, dietary patterns and physical activity do not only differ geographically between populations but differ between children and adults.

From a clinical point of view, investigating the impact of gut-derived uremic toxins in children with CKD is still underappreciated, since early-life exposure in a vulnerable population is likely to affect long-term outcomes, which might differ from those observed in adults. Children have maturing organ functions, and injury from uremic toxins during critical growth and development periods may have lifelong implications for kidney function, cardiovascular disease, and immune maturation [17]. In addition, the cumulative, lifelong exposure to increased uremic toxins levels is highest in children, which reveals the discrepancy between clinical need and scientific knowledge.

## How pediatric CKD cohorts contribute to the field

The CKiD study as well as the cardiovascular comorbidities in children with CKD (4C) study are both multicenter, prospective cohort studies of children and adolescents with mild to moderately impaired kidney function recruited in the USA and Europe, respectively. In line with its longitudinal design and availability of follow-up data, there have been meaningful publications not only on disease etiology and phenotypes but also on risk factors associated with the progression of CKD. In addition, neurocognitive (CKiD) and cardiovascular outcomes (CKiD and 4C) have been assessed.

The CKiD consortium performed untargeted and targeted metabolite quantification based on mass spectrometry. At baseline and during follow-up measurements after 2 and 4 years, they annotated 622 plasma metabolites [2, 18, 19]. Based on this resource, they explored associations between metabolite signatures and the progression of CKD [20] and neurocognitive outcomes [18]. In addition, they used machine learning to identify metabolomic signatures specifically related to different underlying disease etiologies [19].

In the 4C cohort, the prototypic uremic toxins 3-indoxylsulfate (IxS) and p-cresol sulfate (pCS) have been quantified and associated with longitudinally assessed kidney and cardiovascular outcomes [21, 22]. Moreover, there have been smaller pediatric studies investigating the role of these metabolites in the context of growth [23] and kidney replacement therapy [24, 25].

In the current manuscript, Ebrahimi and colleagues describe absolute quantifications of five prototypic gut-derived uremic toxins in a subset of 150 patients in the CKiD study: IxS, pCS, indoleacetate (IA), p-cresol glucuronide (pCG), and phenylacetylglutamine (PAG) [4]. The authors selected these metabolites based on their technical capabilities for absolute quantification and previous research demonstrating their significance in uremic toxicity. IA and IxS are bacterial-derived Trp metabolites, the latter sulfated by the liver. p-cresol is metabolized from tyrosine and glucuronidated to pCG or sulfated to pCS by the liver. PAG is derived from the liver metabolism of microbially produced phenylacetate and was recently shown to activate beta-adrenergic receptors. This manuscript adds some meaningful information to the field and, moreover, highlights persisting gaps of knowledge in the expanding field of microbiome and metabolome analyses in CKD.

First, the absolute quantification of the reported toxins showed a CKD stage-dependent increase, which is in line with what has been reported in adults [26] and children [22, 24] with CKD. However, like previous studies, the current analysis does not allow for more specific speculations about potential factors, beyond CKD stage and etiology that contribute to the variability of toxin levels. Whereas in the 4C study, where serum levels of pCS were higher in patients with CAKUT when compared to glomerular diseases [22], the current study [4] reports lower pCS levels but higher levels of IxS, PAG, and pCG among patients with CAKUT. Therefore, possible associations with the use of antibiotics in distinct patient groups and potentially harmful consequences on the gut microbiome and microbial toxin production and/or absorption are still pending.

Second, both CKiD and 4C explored potential associations between metabolites and clinical outcomes. Prevention of the progression of CKD is of outstanding importance, so a deeper understanding of the underlying mechanisms is crucial for drug development and further improvements of current treatment strategies. In vitro and animal experiments describe the deleterious effects of uremic toxins on kidney damage and fibrosis. Among 622 metabolites annotated in the CKiD cohort, 406 metabolites are associated with eGFR. Despite a relatively moderate mean decline in eGFR of 5 and 7 ml/min after 2 and 4 years, respectively, the change of 35 metabolites was associated significantly with the decline of eGFR [2]. Among those, the level of 6 metabolites (namely N6-carbamoylthreonyladenosine, pseudouridine, 5,6-dihydrouridine, C-glycosyltryptophan, lanthionine, gulonate) were associated independently with a composite endpoint of kidney replacement therapy or 50% reduction of eGFR [20]. None of these metabolites has been validated in the current study by targeted metabolomics. The current study does not report the impact of the quantified metabolites on kidney outcome, whereas in the 4C study, IxS was independently associated with the progression of CKD [21]. There are several possible explanations for why IxS is not significantly related to the progression of CKD in CKiD (untargeted) but in 4C, including technical (untargeted vs*.* targeted approach), statistical (622 vs*.* 2 metabolites) and clinical explanations (eGFR at baseline was 54 in CKiD vs*.* 27 ml/min × 1.73 m^2^ in 4C).

**Fig. 1:**
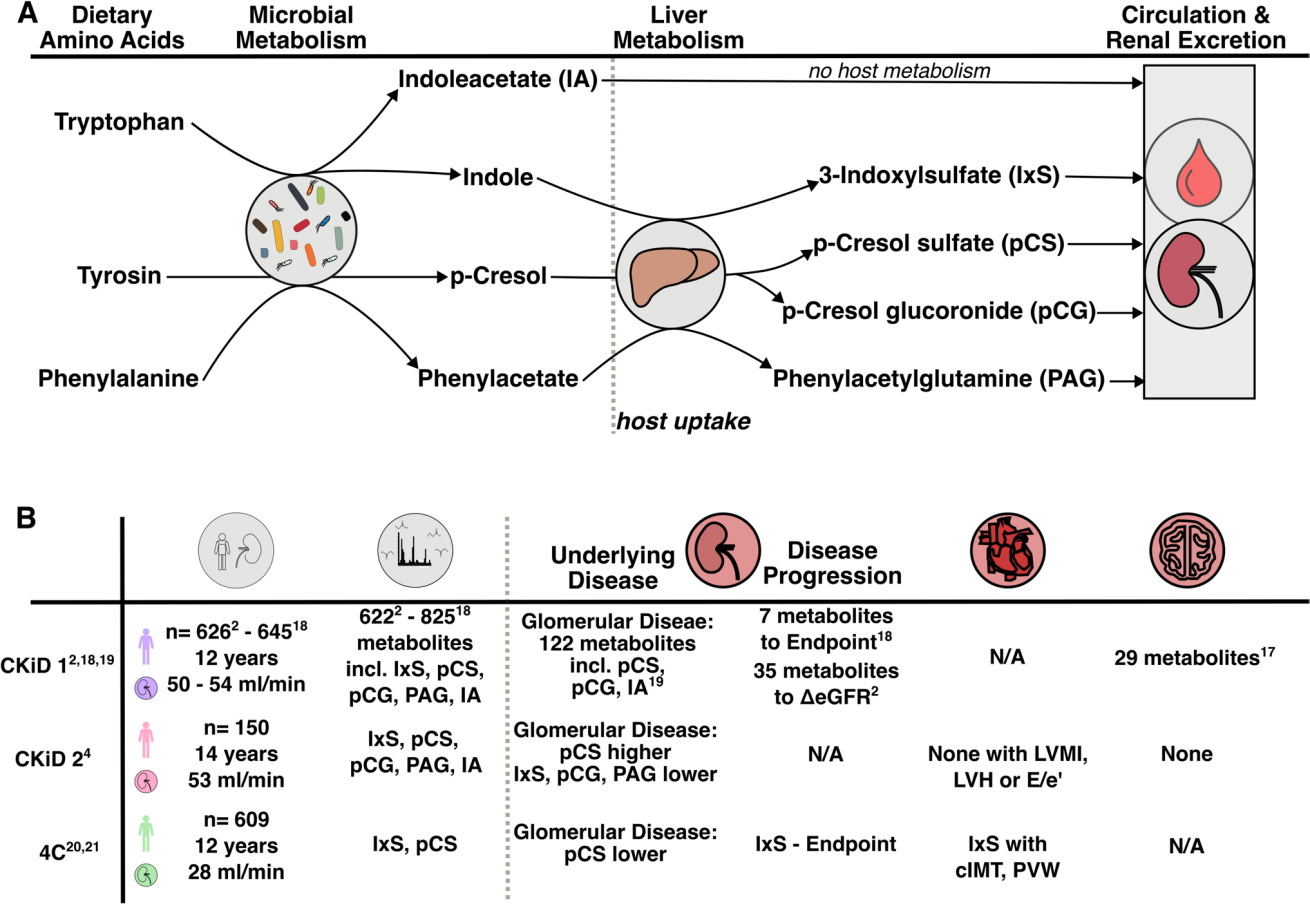
**A** Overview of the origin and metabolism of the metabolites measured in the manuscript of Ebrahimi and colleagues (termed CKiD 2 in this graphic). **B** Overview of key metabolite-outcome associations reported in pediatric CKD in past CKiD manuscripts (CKiD 1), 4C manuscripts, and the current study (CKiD 2)

In addition, the authors of the current study reported neurocognitive and cardiovascular outcomes, showing no significant positive associations with the metabolite levels. Again, this contrasts with what was reported previously in the CKiD study, where both PAG and IA were associated among 27 other metabolites with neurocognitive outcomes [18]. Like the explanations mentioned above, the current analysis only focused on a subgroup of patients from the CKiD cohort, which might explain the discrepancy. Moreover, there was no positive association between reported metabolite levels and left ventricular hypertrophy or mass index. While in the 4C study, there was a positive correlation of IxS with carotid intima-media thickness and progression of pulse wave velocity, these outcome parameters have not yet been investigated by the CKiD consortium, although cIMT is available in a subset of patients [27]. The key findings of metabolite-outcome associations are summarized in Fig. 1.

## Uremic toxins as a target to treat and modify disease outcomes

Taken together, both the CKiD and the 4C cohort report a stage-dependent increase in circulating uremic toxin levels in children, which is consistent with what has been described in adult CKD patients [26, 28]. In adult CKD patients, uremic toxins are clearly associated with gastrointestinal and neurologic symptoms in uremia [29], whereas a heterogeneous correlation with cardiovascular outcomes has been reported, probably due to confounding comorbidities in elderly populations [30–32]. So far, smaller interventional studies targeting the gut microbiome and microbial toxin production in adult populations report conflicting outcomes [33–35]. However, most of these studies only report very limited data on microbiome and metabolome profiles. In contrast, there is increasing knowledge from animal experiments underscoring the pivotal role of uremic toxins in CKD-related pathologies and deciphering the underlying molecular pathways. An example from recent literature about a potential intervention with the prebiotic inulin nicely illustrates some discrepancies between animal and human studies highlighting the need for more translational research: In a rat model of CKD, high-fiber (inulin) diet altered the microbiota, increasing Bifidobacterium and Lactobacillus while decreasing Clostridiaceae and Ruminococcaceae. This was accompanied by decreased levels of IxS and pCS and attenuated aortic calcification, left ventricular hypertrophy, and cardiac fibrosis markers (TGF-β) [36]. In a human pilot study, inulin intervention had similar effects on microbiome composition but failed to lower circulating IxS and pCS levels [37].

Future research needs to focus more on the molecular characterization of distinct subpopulations of CKD patients to better match microbiome-targeted interventions to patients who might benefit from these treatments. Besides clinical parameters, environmental factors need more appreciation, as, for instance, dietary patterns or physical activity have been shown to be crucial for treatment success [38]. The impact of diet has also been highlighted by several editorial comments on the slogan of this year’s World Microbiome Day on June 27: “Feed your microbes – How diet shapes your gut microbiome” [39]. Evidently, the individual patient’s microbiome is key to the success of microbiome-targeted interventions, and efforts are still needed to predict metabolic profiles in the human gut [40]. In addition, diagnostic tools for quick, cheap, and reliable detection of microbiome signatures need to be established in this context to pave the way toward individualized treatments. We should take the chance to investigate the impact of dietary interventions and pre-, pro-, post-, or synbiotic interventions in children, as the risk of side effects is low in comparison to conventional drugs, and treatment response might be better in the absence of multimorbidity and frailty.
